# Al-Doped ZnO Monolayer as a Promising Transparent Electrode Material: A First-Principles Study

**DOI:** 10.3390/ma10040359

**Published:** 2017-03-29

**Authors:** Mingyang Wu, Dan Sun, Changlong Tan, Xiaohua Tian, Yuewu Huang

**Affiliations:** 1 School of Mechanical Engineering, Harbin University of Science and Technology, Harbin 150080, China; 2 College of Applied Science, Harbin University of Science and Technology, Harbin 150080, China; wyysundan@gmail.com (D.S.); xiaohuatian@hrbust.edu.cn (X.T.); 3 School of Materials Science and Engineering, Harbin Institute of Technology, Harbin 150001, China; wuyuehuang@gmail.com

**Keywords:** ZnO monolayer, transparent electrode, electrical conductivity, optical property

## Abstract

Al-doped ZnO has attracted much attention as a transparent electrode. The graphene-like ZnO monolayer as a two-dimensional nanostructure material shows exceptional properties compared to bulk ZnO. Here, through first-principle calculations, we found that the transparency in the visible light region of Al-doped ZnO monolayer is significantly enhanced compared to the bulk counterpart. In particular, the 12.5 at% Al-doped ZnO monolayer exhibits the highest visible transmittance of above 99%. Further, the electrical conductivity of the ZnO monolayer is enhanced as a result of Al doping, which also occurred in the bulk system. Our results suggest that Al-doped ZnO monolayer is a promising transparent conducting electrode for nanoscale optoelectronic device applications.

## 1. Introduction

Transparent conducting oxide (TCO) films have been studied for various photo-electronic devices such as displays, piezoelectric transducers, and solar cells, due to their distinctive combination of optical transparency in the visible spectrum and high electrical conductivity [1,2,3,4]. Currently, Tin doped indium oxide (ITO) is indeed the most widely used TCO in commercial application. However, indium has some disadvantage such as toxicity and high cost, so it is hardly suitable for large-area applications [5]. In recent years, Al-doped ZnO (AZO) has been considered to be one of the best candidates as transparent electrode in thin film solar cells and flat-panel displays due to the lower cost arising from the abundance of Zn and Al, and better optical transmission in the visible region [6].

ZnO material can be applied to light emitting devices, due to the wide band gap (3.37 eV), a high exciton binding energy of 60 meV, and transparency under visible light [7,8]. Unfortunately, ZnO is characterized by a low electrical conductivity, making its use in the photovoltaic field difficult. The doping of different impurity atoms can lead to an improvement in the electrical and optical properties. Saniz et al. demonstrated that the Al substituted Zn site of ZnO is favorable energetically [9]. ZnO doped with group-Ш elements, such as Al and Ga, is regarded as a promising alternative material to ITO, owing to high transparency, low cost, non-toxicity, and high chemical stability [10]. Experimentally, several works show an improvement in the electrical conductivity of ZnO on doping with Al without any significant deterioration in the optical transmittance [11,12,13]. Therefore, Al-doped ZnO as one of the most promising transparent electronics materials has been widely explored.

With the development of the TCO, it has become necessary to achieve high transparency and conductivity in the visible region. The two-dimensional (2D) materials have attracted extensive interest recently due to their unique structure and properties differing from the corresponding bulk materials. After the successful fabrication of graphene in 2004 [14], creating a new research field in 2D materials, an increasing number of other 2D graphene-like materials were successfully realized experimentally [15,16,17,18,19,20,21]. Among these materials, ZnO monolayer has attracted much research interest because of its optoelectronic characteristic [22,23,24,25,26,27,28,29]. Freeman et al. performed density functional calculations to prediction the possibility of forming ultrathin films of wurtzite ZnO with a graphitic structure [22]. Tushce et al. were the first to successfully synthesize two-monolayer-thick ZnO (0001) films deposited on an Ag (111) surface, where the Zn and O atoms are arranged in a planar sheet as in the hexagonal BN monolayer [23]. In a previous study, we explored the structure and optical properties of ZnO monolayer, which were all confirmed as demonstrating potential for future optoelectronic devices [24]. So far, numerous studies have been conducted on Al-doped ZnO in bulk and thin films forms [8,9,10,11,12,13]. However, there have not been any previous investigations on the Al-doped ZnO monolayer in spite of its unique structure and properties differing from its bulk counterpart.

In this work, we systematically investigated the electrical and optical characterization of Al-doped ZnO bulk and monolayer using first-principle calculations. We found that the transparency in the visible light region of Al-doped ZnO monolayer is significantly enhanced compared to the bulk counterpart. Moreover, the electrical conductivity of the ZnO monolayer is enhanced as a result of Al doping, which also occurred in the bulk system. It was therefore concluded that Al-doped ZnO monolayer is a promising transparent conducting electrode for nanoscale optoelectronic device applications.

## 2. Calculation Models and Methods

**Figure 1:**
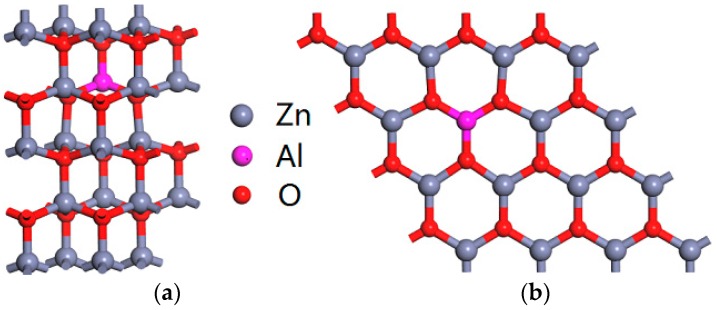
The structures of 6.25 at% Al-doped (**a**) bulk ZnO and (**b**) ZnO monolayer.

All the calculations were performed using CASTEP code based on density functional theory (DFT) [30]. The interaction between ions and electron is described by ultrasoft pseudopotentials [31]. The cutoff energy of 400 eV is used for plane-wave expansion. The Brillouin zone integrations were performed by using a Monkhorst-Pack grid of 4 × 4 × 2 and 6 × 6 × 1 for Al-doped ZnO bulk and monolayer, respectively [32]. The total energy was converged to less than 10^−6^ eV. Structural optimization was performed until the remaining force on each atom was less than 0.02 eV/Å. In order to investigate the different concentrations of the Al atom, the models of 2 × 2 × 2 supercell for bulk wurtzite ZnO and 4 × 4 supercell for ZnO monolayer with one to three Zn atoms replaced by Al atoms were considered. These models correspond to 6.25 at%, 12.5 at% and 18.75 at% of Al in ZnO bulk and monolayer, respectively. For example, Figure 1 shows the structures of 6.25 at% Al in ZnO bulk and monolayer. Moreover, a large vacuum spacing of 15 Å was taken to prevent mirror interactions for ZnO monolayer systems.

The DFT + *U*_d_ + *U*_p_ method was used to describe the electronic structure accurately. Ma et al. suggested that for oxide materials the *U*_p,O_ value of 7 eV is suitable for first principles calculations [33]. Wu et al. showed that when both the *U*_d_ value for Zn 3*d* and the *U*_p_ value for O 2*p* were employed with 10 eV and 7 eV respectively, the calculated band gap and lattice parameter of pristine ZnO bulk are in excellent agreement with experimental values [34]. Therefore, we adopted *U*_d,Zn_ = 10 eV and *U*_p,O_ = 7 eV in this study.

All optical properties can be determined from the dielectric function *ε* of each compound. It describes the optical properties as a function of the frequency or the wavelength. It consists of two parts *ε*_1_ and *ε*_2_:

(1) $$\varepsilon (\omega )=\varepsilon_{1}(\omega )+i\varepsilon_{2}(\omega )$$

where *ε*_1_ and *ε*_2_ are given by [35]:

(2) $$\varepsilon_{2}(\omega )=\frac{4}{\pi \omega^{2}}\sum_{nn^{\prime }}\int {\left|P_{nn^{\prime }}(k)\right|}^{2}\frac{dS_{k}}{\nabla \omega_{nn^{\prime }}(k)}$$

(3) $$\varepsilon_{1}(\omega )=1+\frac{2}{\pi }P\int_{0}^{\infty }\frac{\omega^{\prime }\varepsilon_{2}(\omega^{\prime })}{{\omega^{\prime }}^{2}-\omega^{2}}d\omega^{\prime }$$

$P_{nn^{\prime }}(k)$ is the dipole matrix elements. $\omega_{nn^{\prime }}(k)$ is the energy difference between initial and final states. *S_k_* denotes the surface energy and *P* is the principal part of the integral. The coefficient of absorption *α* is proportional to the imaginary part *ε*_2_:

(4) $$\alpha (\omega )=\frac{\omega \varepsilon_{2}(\omega )}{c}$$

the refractive index n(ω) is a function of *ε*_1_ and *ε*_2_:

(5) $$n(\omega )=\frac{1}{\sqrt{2}}{\left[{\left(\varepsilon_{1}^{2}(\omega )+\varepsilon_{2}^{2}(\omega )\right)}^{\frac{1}{2}}+\varepsilon_{1}(\omega )\right]}^{1/2}$$

the reflectivity R(ω) can be given by [36,37]:

(6) $$R(\omega )=\left|\frac{\tilde{n}-1}{\tilde{n}+1}\right|=\frac{{\left(n-1\right)}^{2}+k^{2}}{{\left(n+1\right)}^{2}+k^{2}}$$

To calculate the electrical properties of the pristine and Al-doped bulk and monolayer ZnO systems, we fitted the calculated band structure into a Boltzmann package, which is based on semi-classic Boltzmann theory and the rigid band approach [38,39]. The dependence of the conductivity on transport distribution can be given by:

(7) $$\sigma_{\alpha \beta }\left(\varepsilon \right)=\frac{1}{N}\sum_{i,k}\sigma_{\alpha \beta }\left(i,k\right)\frac{\delta \left(\varepsilon -\varepsilon_{i,k}\right)}{\delta \left(\varepsilon \right)}$$

where, *N* denotes the number of *k*-points that are sampled in the Brillouin zone and *ε_i,k_* presents the band structure. The *k*-dependent transport tensor is read as:

(8) $$\sigma_{\alpha \beta }\left(i,k\right)=e^{2}\tau_{i,k}v_{\alpha }\left(i,k\right)v_{\beta }\left(i,k\right)$$

with, *i* and *k* stand for the band index and wave vector, respectively, and *τ* denotes the relaxation time, *ν_α_*(*i*,*k*) is the *α* component of the group velocities, while *e* is the electron charge.

By fitting the transport distribution over energy, the electrical conductivity can be obtained as a function of the temperature, *T*, and the chemical potential, *μ*, via the following equations:

(9) $$\sigma_{\alpha \beta }\left(T,\mu \right)=\frac{1}{\Omega }\int \sigma_{\alpha \beta }\left[-\frac{\partial f_{\mu }\left(T,\varepsilon \right)}{\partial \varepsilon }\right]d\varepsilon$$

where *α* and *β* stand for the tensor indices, Ω, *μ*, and *f* denote the volume of the unit cell, the Fermi level of carriers, and the carrier Fermi-Dirac distribution function, respectively.

Owing tothe complexity of the electron scattering mechanisms in the material, the exact solution of the Boltzmann equation cannot be achieved. To overcome this issue, the relaxation time is treated as an energy-independent constant, which is known as the relaxation time approximation. This approach has been demonstrated to be a reasonable good approximation for evaluating the electrical transport properties of several materials [40,41,42].

## 3. Results and Discussion

### 3.1. Structural Properties

Firstly, we explored the structural properties of Al-doped ZnO bulk and monolayer. Table 1 shows the lattice parameters for the optimized structure of the studied systems at various Al concentrations. The lattice parameters of pristine bulk ZnO are *a* = 3.313 Å and *c* = 5.329 Å, which are close to the experimental values of *a* = 3.249 Å and *c* = 5.206 Å [43] and in good agreement with other theoretical calculations [44], indicating that our calculations are reliable. Starting from a structure cut from the ZnO wurtzite crystal and terminating with the (0001) polar surface, atomic relaxations from the DFT calculations indicate that ZnO monolayer stabilizes in a hexagonal BN structure. The calculated Zn-O bond length in the ZnO monolayer is 1.91 Å, which is lower than the corresponding bond length in bulk ZnO (2.01 Å). This is due to the fact that the *sp*^2^ hybridization in the 2D honeycomb structure is stronger than the *sp*^3^ hybridization in wurtzite crystal. Moreover, it is found that the lattice constant *a* of Al-doped ZnO monolayer decreases with increasing Al concentration. This phenomenon is also observed in bulk ZnO. The reason is attributed to the fact that the radius of Al atom is smaller than that of Zn.

The formation energy is an important parameter to describe material stability. It is well known that material systems with lower formation energies are more stable than those with higher formation energies. The negative formation energy means the formation of the structure is thermodynamically favorable. To evaluate the plausibility of doping Al atoms in ZnO bulk and monolayer, we calculated their formation energies, as shown in Table 1. The definition of formation energy was given in our previous work [24]. From Table 1, it can be seen that formation energies of both Al-doped ZnO bulk and monolayer are found to be negative, signifying that Al atoms are suitable for doping into ZnO systems. More interestingly, we found that the value of the formation energy for the Al-doped ZnO monolayer is lower than that of the bulk counterpart at the same Al concentration. We can conclude that the preparation of Al-doped ZnO monolayer could be rather easily achieved in experiments, because Al-doped ZnO bulk has been successfully prepared. Our results of formation energies would be useful for designing the growth process and application of Al-doped ZnO monolayer.

### 3.2. Electronic Properties

**Figure 2:**
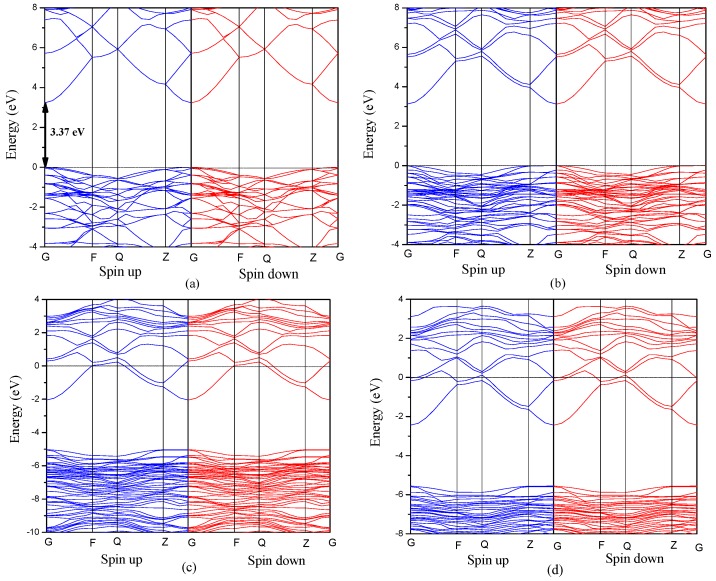
The band structures of (**a**) pristine ZnO bulk; (**b**) 6.25 at%; (**c**) 12.5 at%; (**d**) 18.75 at% Al-doped ZnO bulk.

**Figure 3:**
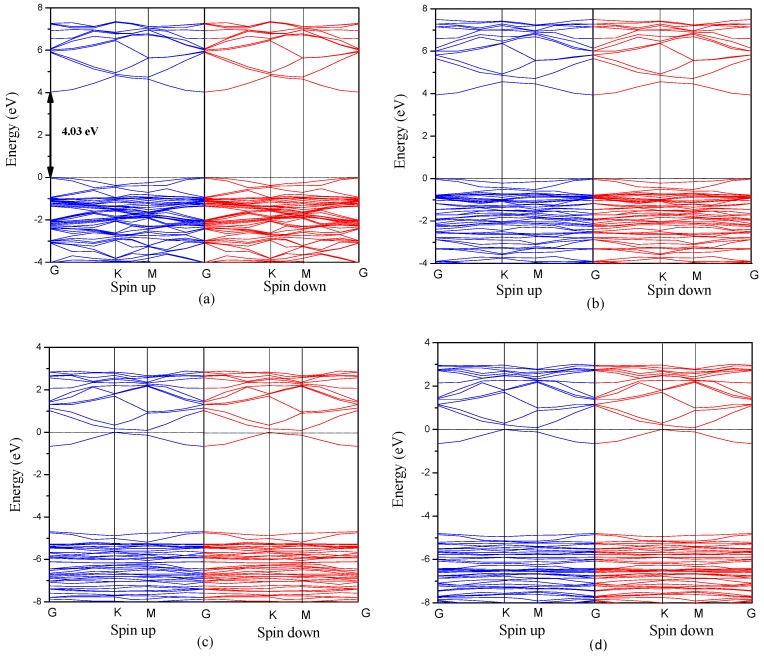
The band structures of (**a**) pristine ZnO monolayer; (**b**) 6.25 at%; (**c**) 12.5 at%; (**d**) 18.75 at% Al-doped ZnO monolayer.

Next, we turn to the effect of Al doping on the electronic properties of ZnO bulk and monolayer, where we calculated the band structure of Al-doped ZnO bulk and monolayer as shown in Figure 2 and Figure 3, respectively. From Figure 2a, the calculated band structure shows that pristine ZnO bulk is a direct band gap semiconductor with the conduction band minimum (CBM) and the valence band maximum (VBM) located at the same G point of the Brillouin zone. The VBM orbitals are mainly due to Zn 3*d* states and the CBM orbitals are mainly from O 2*p* states. The corresponding band gap is 3.37 eV, which is in good agreement with the experiment [45]. When the Al concentration in ZnO bulk is large than 12.5 at%, the Fermi level shifts upward into the conduction band as seen in Figure 2c,d, which indicates that this sample is an n-type semiconductor. It means that shallow donor states are created around the Fermi level in the bottom of the conduction band, leading to the increase of carrier concentration.

From Figure 3a, it is seen that the direct band gap of pristine ZnO monolayer (4.03 eV) is much larger than that of bulk material due to the quantum confinement effect. Moreover, from Figure 3b–d, it can be seen that the band structure of Al-doped ZnO monolayer is similar to that of Al-doped ZnO bulk. The Fermi level also moves into the conduction band when the Al concentration is large than 12.5 at%.

**Figure 4:**
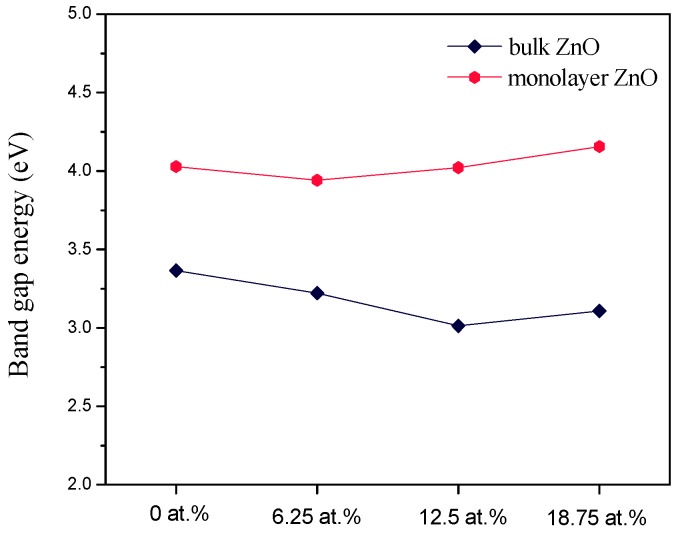
The band gap of Al-doped ZnO bulk and monolayer.

Furthermore, the band gaps of Al-doped ZnO bulk and monolayer as a function of Al concentration are shown in Figure 4. For the bulk ZnO structure, the band gap decreases with increasing Al concentration at the range of less than 12.5 at%. The reason lies in the fact that the conduction band moves to the low-energy region as Al atoms are incorporated into bulk ZnO, leading to the decrease of band gap. However, when the Al concentration is higher than 12.5 at%, the band gap increases slightly with increasing Al concentration. In terms of Al-doped ZnO monolayer, the band gap decreases first when the Al concentration is less than 6.25 at%, and then increases with further increase in Al concentration. Particularly, it is observed that the band gap of Al-doped ZnO monolayer is obviously larger than that of ZnO bulk counterpart. Consequently, it is to be expected that the band-edge absorption of Al-doped ZnO monolayer could be blue shifted with increasing Al concentration compared to the Al-doped ZnO bulk counterpart.

**Figure 5:**
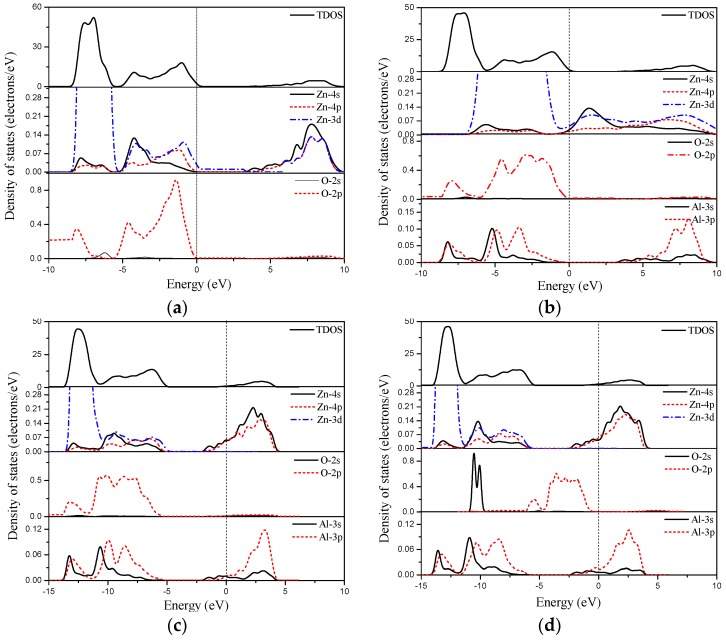

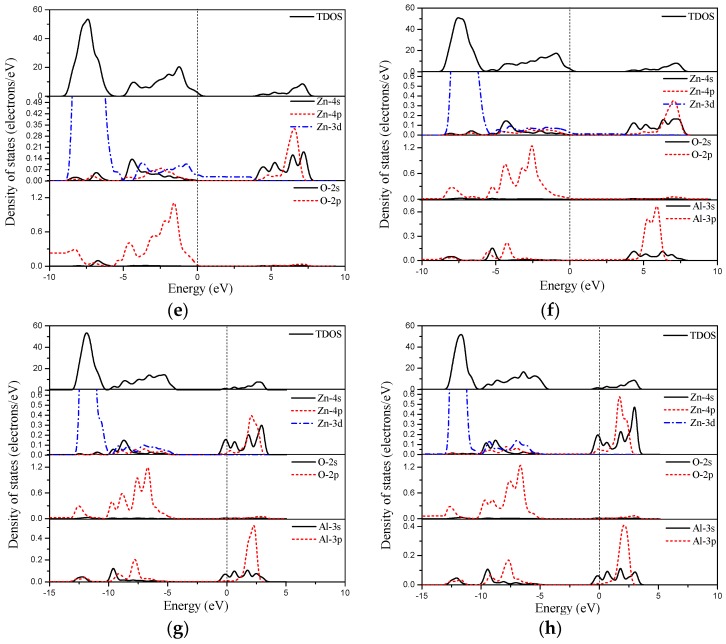
The total and partial total density of states (DOS) of (**a**) pristine bulk ZnO; (**b**) 6.25 at%; (**c**) 12.5 at%; (**d**) 18.75 at% Al-doped bulk ZnO; (**e**) pristine ZnO monolayer; (**f**) 6.25 at%; (**g**) 12.5 at%; (**h**) 18.75 at% Al-doped ZnO monolayer.

To further investigate the effect of Al doping on the conductance in detail, Figure 5 presents the total density of states (DOS) of ZnO bulk and monolayer doped with different Al concentrations. The results obviously indicate that Al doping makes the Fermi level of ZnO monolayer enter the conduction band when the Al concentration is large than 12.5 at%. This phenomenon occurred in the Al-doped ZnO bulk system as well. Upon Al doping, electrons are introduced into the conduction band of ZnO monolayer, which increases the concentration of free electrons, resulting in the decrease in the electronic resistance of Al-doped ZnO bulk and monolayer. The occupied states of electrons in the conduction band near the Fermi level are generally related to the donor concentration. From Figure 5, it can be seen that the Al 3*s* orbital contributes to the occupied states around the Fermi level. These donor states around the Fermi level could be considered as the origin of the conductivity increase in the Al doped ZnO sample. Furthermore, we find that the donor states extend gently and deeply into the conduction band with increasing Al concentration, thus the conductance of both Al-doped ZnO bulk and monolayer is significantly enhanced. Therefore, less energy is required to liberate electrons from the material, resulting in the increase in conductance. The enhanced conductance of ZnO monolayer due to Al doping, which is similar to the doping effect in ZnO bulk, provides a basis for its potential application as a transparent electrode.

### 3.3. Optical Properties

**Figure 6:**
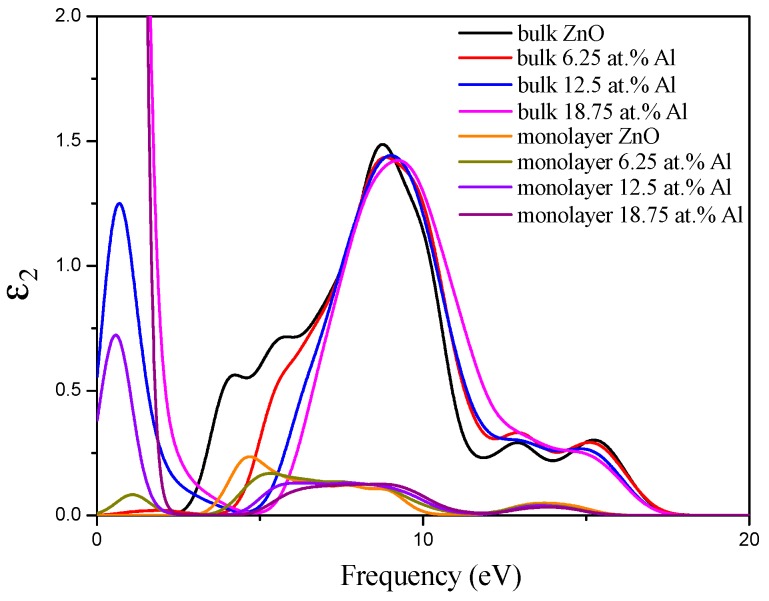
The imaginary part of the dielectric function of ZnO bulk and monolayer doped with different Al concentrations.

In this section, we present and compare the calculated optical properties of pristine and different concentrations of Al-doped ZnO bulk and monolayer. It is well known that the good TCOs should have low absorption and reflectivity coefficient with large transmittance in the large wavelength region. In order to describe the optical properties of these structures, we calculated the imaginary part of the dielectric function through the formulas mentioned in the above section of calculation methods. The reflectivity, the absorption coefficient, and the transmittance can be calculated from the dielectric function. The imaginary parts of the dielectric functions of ZnO bulk and monolayer doped with different Al concentrations were calculated and are compared in Figure 6. From this figure, we can see the imaginary part of the dielectric function of Al-doped ZnO bulk is smaller than that of pristine ZnO bulk, and has a blue shift to the higher energy side. This result is consistent with a previous study [46]. Moreover, the main peak width of the imaginary part of the dielectric function narrows following increasing Al concentration, indicating that the range of absorption frequency narrows and the average optical transmittance increases. On the other band, for the Al-doped ZnO monolayer, the imaginary part of the dielectric function is much smaller than that of Al-doped ZnO bulk. The main peak at about 4.69 eV of pristine ZnO monolayer moves to the higher energy side after Al doping. Moreover, the energy value of this main peak decreases with increasing Al concentration. In addition, after the 6.25 at% Al is doped into the ZnO monolayer, a new peak is formed at low energy, due to the transition between the Al-3*s* donor occupied states around the Fermi level and the unoccupied Zn-4*s* and Zn-4*p* states in the conduction band. The new peak is enhanced and shifts to lower energy on increasing the Al concentration to 12.5 at%. Furthermore, this peak becomes intense when the Al-doped concentration increases to 18.75 at%. The reason may be that doping of high Al concentration results in the occupied states widening.

**Figure 7:**
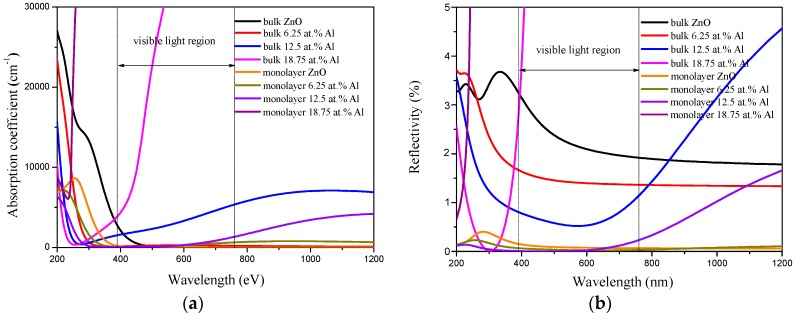

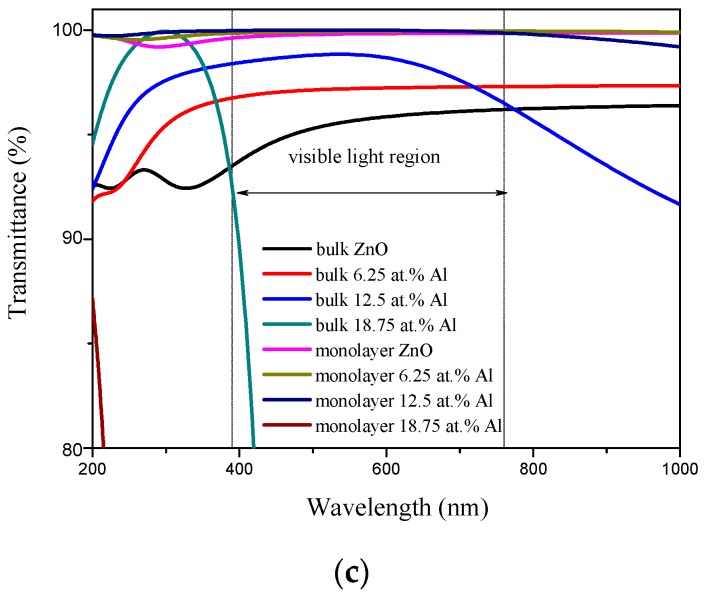
(**a**) Absorption coefficient; (**b**) reflectivity; (**c**) transmittance of Al-doped ZnO bulk and monolayer.

Furthermore, In Figure 7a, we show the absorption coefficient as a function of wavelength for pristine and Al-doped ZnO bulk and monolayer. It is clear that the pristine ZnO bulk has a low absorption coefficient in the visible and infrared (IR) regions. Moreover, the absorption coefficient increases from the visible to ultraviolet (UV) regions. When the Al is doped in ZnO bulk, the absorption coefficient decreased and shifted to the lower wavelength region in the visible and UV regions. When the Al concentration is 12.5 at%, the absorption coefficient slowly increased in the range of 276 nm to 1070 nm. Meanwhile, it can be observed that the shallow donor states induce increasing absorption in the visible region for 18.75 at% Al-doped bulk ZnO. For the pristine ZnO monolayer, the optical absorption coefficient calculated between 200 nm and 1200 nm is lower than that of ZnO bulk in the UV and visible regions, and presents a decline at about 255 nm. When the Al is doped, the optical absorption edge of ZnO monolayer has a clear blue-shift to a shorter wavelength region with increasing Al concentration. Meanwhile, following the increase of Al concentration, the absorption coefficient decreases and becomes small in the visible region. This is due to the fact that Al doping decreases the concentration of Zn, resulting in a decrease of absorption. However, the absorption coefficient of Al-doped ZnO monolayer increases in the range from 600 nm to 1200 nm. Particularly, it is worth noting that the absorption coefficient obviously increases in the range of 800 nm to 1200 nm when the Al concentration is 12.5 at%.

The reflectivity and transmittance are important parameters to describe the optical property of TCOs. Figure 7b,c show the reflectivity and transmittance of Al-doped ZnO bulk and monolayer, respectively. The reflectivity of pristine ZnO bulk is low in the visible region and the average transmittance of pristine ZnO bulk is around 94%. For Al-doped ZnO bulk, the average transmittance increases with Al concentration in the UV and visible regions. This case is also reported in many experimental studies concerning Al-doped ZnO thin films [47]. The conventional ITO electrode has a transmittance of about 80% [48]. Therefore, Al-doped ZnO bulk is a high transparency material and has promise for transparent electronics. Nevertheless, when the Al concentration reaches 18.75 at%, the reflectivity rises rapidly and the transmittance declines rapidly at 300 nm due to the high carrier concentration.

For the pristine ZnO monolayer, the reflectivity is obviously lower and the transmittance is far larger than that of bulk ZnO, as shown in Figure 7b,c. When Al is doped, the reflectivity of ZnO monolayer decreases with increasing Al concentration in the visible and UV regions. However, the reflectivity slowly increases at 560 nm for Al-doped ZnO monolayer at 12.5 at% Al concentration. When the Al concentration is at 18.75 at%, the reflectivity speed increases in the UV region. The average transmittance of 6.25 at% Al-doped ZnO monolayer reaches 98% in the visible range (390–760 nm). When the Al concentration is 12.5 at%, the highest visible transmittance of about 99% is achieved. However, when the Al concentration is 18.75 at%, the transmittance of the Al-doped ZnO monolayer rapidly decreases in the UV region. Overall, the transparency in the visible light region of the Al-doped ZnO monolayer is significantly enhanced compared to that of bulk counterpart. In particular, 12.5 at% Al-doped ZnO monolayer transmits more light.

### 3.4. Transport Properties

Here, we investigated the electrical transport properties in the pristine and Al doped ZnO bulk and monolayer using the Boltzmann transport equations. We calculated the electrical conductivity *σ*/*τ* of pristine and Al-doped ZnO bulk and monolayer as a function of the relaxation time *τ* at room temperature. However, one of the major shortcoming of calculating the *σ* value is the knowledge of the relaxation time relation. To overcome this shortcoming, we used the relaxation time relationship reported by Ong et al. [49] using the same method as that for ZnO material. The relationship of relaxation time is given as following:

(10) $$\tau =2.53\times 10^{-5}T^{-1}n^{-1/3}$$

where *T* is the temperature and *n* is the electron concentration.

**Figure 8:**
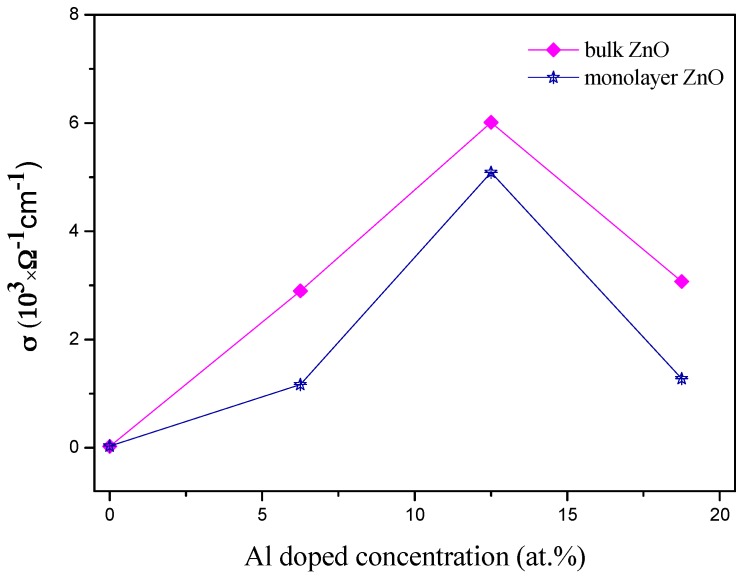
The electrical conductivity of ZnO bulk and monolayer doped with different Al concentrations.

First, we fixed the temperature at 300 K (room temperature), taking the electron concentration from our calculation for pristine and Al-doped bulk and monolayer ZnO. We obtained, from this relationship, the estimated values of relaxation times. Then we used these relaxation times to calculate the electrical conductivity *σ* as (*σ/τ*) × *τ*. The obtained values of the electrical conductivity are shown in Figure 8. From this figure, we find that the electrical conductivity obviously increases with increasing Al concentration in both bulk and monolayer ZnO when the Al concentration is less than 12.5 at%. However, for the higher concentration of Al, the electrical conductivity decreased. By numerical comparison, after Al doping, the electrical conductivity of ZnO monolayer is slightly lower than that of bulk material. However, for the monolayer material, this value is already relatively high. Therefore, combining the results of optical and transport properties, we suggest that Al-doped ZnO monolayer would be promising transparent electrode material for nanoscale optoelectronic device applications.

## 4. Conclusions

In summary, we studied the electronic structure and optical properties of Al-doped ZnO bulk and monolayer. We found that the band gap of the pristine and Al-doped ZnO monolayer is larger than that of the bulk system due to the quantum confinement effect. In addition, after Al is doped in the ZnO monolayer, the Fermi level shifts into the conduction band and there is a shallow donor state around the Fermi level. Moreover, the transparency in the visible light region of Al-doped ZnO monolayer is significantly enhanced compared to bulk counterpart. In particular, the 12.5 at% Al-doped ZnO monolayer exhibits the highest visible transmittance of above 99%. Furthermore, our calculations show that the electrical conductivity of ZnO monolayer is enhanced by Al doping and exhibits a relatively high value. These results show that Al-doped ZnO monolayer is a promising transparent conducting electrode for nanoscale optoelectronic device applications.

## Figures and Tables

**Table 1:** The lattice parameters and formation energies of Al-doped ZnO bulk and monolayer.

Compounds	Concentration	*a* (Å)	*c* (Å)	Formation Energy (eV)
bulk	0%	3.313	5.329	-
	6.25%	3.312	5.315	−0.096
	12.5%	3.307	5.320	−0.162
	18.75%	3.306	5.324	−0.231
monolayer	0%	4.437	-	-
	6.25%	4.426	-	−0.059
	12.5%	4.421	-	−0.135
	18.75%	4.408	-	−0.199

## References

[1] Kim Y.S., Hwang W.J., Eun K.T., Choa S.H. (2011). Mechanical reliability of transparent conducting IZTO film electrodes for flexible panel displays. Appl. Surf. Sci..

[2] Wu Z.H., Chen Z.H., Du X., Logan J.M., Sippel J., Nikolou M., Kamaras K., Reynolds J.R., Tanner D.B., Hebard A.F. (2004). Transparent, conductive carbon nanotube films. Science.

[3] Wang X., Zhi L., Müllen K. (2008). Transparent, conductive graphene electrodes for dye-sensitized solar cells. Nano Lett..

[4] Major S., Kumar S., Bhatnagar M., Chopra K.L. (1986). Effect of hydrogen plasma treatment on transparent conducting oxides. Appl. Phys. Lett..

[5] Minami T. (2005). Transparent conducting oxide semiconductors for transparent electrodes. Semicond. Sci. Technol..

[6] Wilken S., Wilkens V., Scheunemann D., Nowak R.E., Maydell K.V., Parisi J., Borchert H. (2015). Semitransparent Polymer-Based Solar Cells with Aluminum-Doped Zinc Oxide Electrodes. ACS Appl. Mater. Interfaces.

[7] Vijayalakshmi K., Karthick K. (2013). Influence of Mg doping on the microstructure and PL emission of wurtzite ZnO synthesized by microwave processing. J. Mater. Sci..

[8] Leonardo G.C., Gontijo C., Alfredo A.P.N. (2012). Electrical, optical, and structural properties of thin films with tri-layers of AZO/ZnMgO/AZO grown by filtered vacuum arc deposition. Mater. Sci. Eng. B.

[9] Saniz R., Xu Y., Matsubara M., Amini M.N., Dixit H., Lamoen D., Partoens B. (2013). A simplified approach to the band gap correction of defect formation energies: Al, Ga, and In-doped ZnO. J. Phys. Chem. Solids.

[10] Hagendorfer H., Lienau K., Nishiwaki S., Fella C.M., Kranz L., Uhl A.R., Jaeger D., Luo L., Gretener C., Buechler S. (2014). Highly transparent and conductive ZnO: Al thin films from a low temperature aqueous solution approach. Adv. Mater..

[11] Crossay A., Buecheler S., Kranz L., Perrenoud J., Fella C.M., Romanyuk Y.E., Tiwari A.N. (2012). Spray-deposited Al-doped ZnO transparent contacts for CdTe solar cells. Sol. Energy Mater. Sol. Cells.

[12] Kang D.W., Kuk S.H., Ji K.S., Lee H.M., Han M.K. (2012). Effects of ITO precursor thickness on transparent conductive Al doped ZnO film for solar cell applications. Sol. Energy Mater. Sol. Cells.

[13] Li J.Z., Xu J., Xu Q.B. (2012). Preparation and characterization of Al doped ZnO thin films by sol–gel process. J. Alloys Compd..

[14] Novoselov K.S., Geim A.K., Morozov S.V., Jiang D., Zhang Y., Dubonos S.V., Grigorieva I.V., Firsov A.A. (2004). Electric field effect in atomically thin carbon films. Science.

[15] Tang Q., Zhou Z. (2013). Graphene-analogous low-dimensional materials. Prog. Mater. Sci..

[16] Xu M., Liang T., Shi M., Chen H. (2013). Graphene-like two-dimensional materials. Chem. Rev..

[17] Koski K.J., Cui Y. (2013). The new skinny in two-dimensional nanomaterials. ACS Nano.

[18] Song X., Hu J., Zeng H. (2013). Two-dimensional semiconductors: Recent progress and future perspectives. J. Mater. Chem. C.

[19] Miro P., Audiffred M., Heine T. (2014). An atlas of two-dimensional materials. Chem. Soc. Rev..

[20] Gupta A., Sakthivel T., Seal S. (2015). Recent development in 2D materials beyond graphene. Prog. Mater. Sci..

[21] Butler S.Z., Hollen S.M., Cao L.Y. (2013). Progress, challenges, and opportunities in two-dimensional materials beyond graphene. ACS Nano.

[22] Freeman C.L., Claeyssens F., Allan N.L., Harding J.H. (2006). Graphitic nanofilms as precursors to wurtzite films: Theory. Phys. Rev. Lett..

[23] Tusche C., Meyerheim H.L., Kirschner J. (2007). Observation of depolarized ZnO(0001) monolayers: Formation of unreconstructed planar sheets. Phys. Rev. Lett..

[24] Tan C.L., Sun D., Xu D.S., Tian X.H., Huang Y.W. (2016). Tuning electronic structure and optical properties of ZnO monolayer by Cd doping. Ceram. Int..

[25] Weirum G., Barcaro G., Fortunelli A., Weber F., Schennach R., Surnev S., Netzer F.P. (2010). Growth and Surface Structure of Zinc Oxide Layers on a Pd(111) Surface. J. Phys. Chem. C.

[26] Quang H.T., Bachmatiuk A., Dianat A., Ortmann F., Zhao J., Warner J.H., Eckert J., Cunniberti G., Rummeli M.H. (2015). In situ observations of free-standing graphene-like mono- and bilayer ZnO membranes. ACS Nano.

[27] Sahoo T., Nayak S.K., Chelliah P., Raht M.K., Parida B. (2016). Observations of two-dimensional monolayer zinc oxide. Mater. Res. Bull..

[28] Tu Z.C. (2010). First-principles study on physical properties of a single ZnO monolayer with graphene-like structure. J. Comput. Theor. Nanosci..

[29] Guo H.Y., Zhao Y., Lu N., Kang E.J., Zeng X.C., Wu X.J., Yang J.L. (2012). Tunable Magnetism in a Nonmetal-Substituted ZnO Monolayer: A First-Principles Study. J. Phys. Chem. C.

[30] Segall M.D., Lindan P.J.D., Probert M.J., Pickard C.J., Hassnip P.J., Clark S.J., Payne M.D. (1990). First-principles simulation: Ideas, illustrations and the CASTEP code. J. Phys. Condens. Matter..

[31] Vanderbilt D. (1990). Ultrasoft pseudopotentials in a generalized eigenvalue formalism. Phys. Rev. B.

[32] Monkhorst H.J., Pack J.D. (1976). Special points for Brillouin-zone integrations. Phys. Rev. B.

[33] Ma X., Lu B., Li D., Shi R., Pan C., Zhu Y. (2011). Origin of photocatalytic activation of silver orthophosphate from first-principles. J. Phys. Chem. C.

[34] Wu H.C., Peng Y.C., Chen C.C. (2013). Effects of Ga concentration on electronic and optical properties of Ga-doped ZnO from first principles calculations. Opt. Mater..

[35] Wooten F. (1972). Optical Properties of Solids.

[36] Sun J., Wang H., He J., Tian Y. (2005). Ab initio investigations of optical properties of the high-pressure phases of ZnO. Phys. Rev. B.

[37] Fox M. (2001). Optical Properties of Solids. Oxford Master Series in Condensed Matter Physics.

[38] Madsen G.K.H., Singh D.J. (2006). A code for calculating band-structure dependent quantities. Comput. Phys. Commun..

[39] Ziman J.M. (2001). Electrons and Phonons.

[40] Gao X., Uechara K., Klug D., Patchkovskii S., Tse J., Tritt T. (2005). Theoretical studies on the thermopower of semiconductors and low-band-gap crystalline polymers. Phys. Rev. B.

[41] Chaput L., Pécheur P., Tobola J., Scherrer H. (2005). Transport in doped skutterudites: Ab initio electronic structure calculations. Phys. Rev. B.

[42] Madsen G.K.H. (2006). Automated search for new thermoelectric materials: The case of LiZnSb. J. Am. Chem. Soc..

[43] Vispute R.D., Talyansky V., Choopun S., Sharma R.P., Venkatesan T., He M., Salamanca-Riba L.G. (1998). Heteroepitaxy of ZnO on GaN and its implications for fabrication of hybrid optoelectronic devices. Appl. Phys. Lett..

[44] Zhang H.F., Lu S.X., Xu W.G., Yuan F. (2014). First-principles study of Si atoms adsorbed on ZnO (0001) surface and the effect on electronic and optical properties. Surf. Sci..

[45] Ozgur U., Alivov Y.I., Liu C., Teke A., Reshchikov M.A., Dogan S., Avrutin V., Cho S.J., Morkoc H. (2005). A comprehensive review of ZnO materials and devices. J. Appl. Phys..

[46] Zhang F.C., Zhang Z.Y., Zhang W.H., Yan J.F., Yun J.N. (2009). First-principles calculation of electronic structure and optical properties of AZO(ZnO:Al). Acta Opt. Sin..

[47] Wang M., Lee K.E., Hahn S.H. (2007). Optical and photoluminescent properties of sol-gel Al-doped ZnO thin films. Mater. Lett..

[48] Seki S., Ogawa M., Sawada Y. (2001). Indium–Tin–Oxide thin films prepared by dip coating: Dependence of resistivity on film thickness and annealing atmosphere. Jpn. J. Appl. Phys..

[49] Ong K.P., Singh D.J., Wu P. (2011). Analysis of the thermoelectric properties of n-type ZnO. Phys. Rev. B.

